# Dual Interferometric Interrogation for DFB Laser-Based Acoustic Sensing

**DOI:** 10.3390/s25092873

**Published:** 2025-05-02

**Authors:** Mehmet Ziya Keskin, Abdulkadir Yentur, Ibrahim Ozdur

**Affiliations:** Department of Electrical and Electronics Engineering, TOBB University of Economics and Technology, Ankara 06510, Türkiye; ayentur@etu.edu.tr (A.Y.); iozdur@etu.edu.tr (I.O.)

**Keywords:** acoustic sensing, fiber lasers, fiber-optic sensors, hydrophones, interferometers

## Abstract

Acoustic sensing has many applications in engineering, one of which is fiber-optic hydrophones (FOHs). Conventional piezoelectric hydrophones face limitations related to size, electromagnetic interference, corrosion, and narrow operating bandwidth. Fiber-optic hydrophones, particularly those employing distributed feedback (DFB) lasers, offer a compelling alternative due to their mechanical flexibility, resistance to harsh conditions, and broad detection range. DFB lasers are highly sensitive to external perturbations such as temperature and strain, enabling the precise detection of underwater acoustic signals by monitoring the resultant shifts in lasing wavelength. This paper presents an enhanced interrogation mechanism that leverages Mach–Zehnder interferometers to translate wavelength shifts into measurable phase deviations, thereby providing cost-effective and high-resolution phase-based measurements. A dual interferometric setup is integrated with a standard demodulation algorithm to extend the dynamic range of these sensing systems. The experimental results demonstrate a substantial improvement in performance, with the dynamic range increasing from 125 dB to 139 dB at 1 kHz without degrading the noise floor. This enhancement significantly expands the utility of FOH-based systems in underwater environments, supporting applications such as underwater surveillance, submarine communication, and marine ecosystem monitoring.

## 1. Introduction

Acoustic sensing is an important branch of engineering that underpins a variety of applications, including military operations, seismic studies, and geoscience research [1,2,3]. Underwater acoustic sensing is one of the most important applications of acoustic sensors. Hydrophones function as specialized acoustic sensing elements optimized for the challenges of underwater environments. Acoustic transducers used in various applications combine mechanics, electronics, optics, magnetics, semiconductors, and acoustics to convert incoming acoustic signals into measurable outputs [2]. Similarly, many commercial hydrophones utilize piezoelectric materials, taking advantage of the piezoelectric effect to offer high manufacturability, low cost, adequate sensitivity, and a sufficiently wide dynamic range. Additionally, some systems incorporate MEMS- and fiber-optic-based hydrophones [2,3,4].

Despite their advantages, conventional piezoelectric hydrophones face several limitations. They tend to be large, are susceptible to electromagnetic interference, and can corrode in harsh environments. Specialized protective coatings are needed for high hydrostatic pressures, reducing efficiency, and these devices are prone to circuit malfunctions, especially underwater. They are also fragile under high pressures, have limited cable lengths due to high electrical impedance, and operate within a relatively narrow frequency bandwidth. Moreover, their sensitivity is tied to their resonant frequency, they have poor multiplexing capabilities, and they are unsuitable for extreme conditions such as high temperatures, high voltages, intense electromagnetic fields, and explosive environments [5,6,7,8].

Fiber-optic-based methods for underwater acoustic sensing have been investigated since the 1970s [4,9]. Fiber-optic hydrophones (FOHs) can offer significant benefits over conventional hydrophones, including simpler structures, lighter weight, greater mechanical flexibility, improved durability under high acoustic pressures, and reduced susceptibility to mechanical and thermal damage [10]. Two primary approaches to fiber-optic acoustic sensing exist: mandrel-based and distributed feedback (DFB) fiber laser-based techniques [6]. Mandrel-based sensing uses a mechanical structure around which a fiber carrying fiber Bragg gratings (FBGs) is tightly wound. Acoustic signals deform the mandrel, thereby straining the fibers and altering the FBGs’ characteristics. In contrast, the DFB fiber-laser-based method—the focus of this study—employs a simpler and more compact sensor design thanks to its thin mechanical structure, which paves the way for a smaller sensor size [10,11,12,13,14].

This study aims to enhance the interferometric demodulation and interrogation mechanisms in underwater acoustic sensing systems that use DFB sensor lasers. DFB sensor lasers are particularly well-suited for detecting underwater acoustic signals in diverse conditions thanks to their broad detection range, narrow linewidths, and remote-operating capabilities. Although their lasing frequency is highly sensitive to external factors such as temperature fluctuations and mechanical strain on the optical fiber, this sensitivity can be harnessed to detect acoustic signals [10]. Shifts in the lasing wavelength, induced by such perturbations, enable the measurement of acoustic signals through wavelength and frequency shifts, which is especially advantageous for long-distance sensing in challenging environments.

To carry out these measurements, Mach–Zehnder or Michelson interferometers are typically employed at the interrogator end to convert wavelength shifts into phase shifts. Phase-based measurements offer several benefits over direct wavelength measurements, including cost-effectiveness, ease of implementation, and higher resolution [9,15]. These advantages make interferometric approaches particularly attractive for underwater applications that require high precision and reliability. However, a key challenge in underwater acoustic sensing remains: preserving a wide dynamic range without sacrificing measurement accuracy or signal integrity. Detecting both weak and strong acoustic signals without distortion is crucial for applications in underwater surveillance, submarine communication, and marine ecosystem monitoring.

Previous research has proposed various methods to evaluate and expand the dynamic range of such systems, often concentrating on algorithmic enhancements or comparative simulations [16,17,18]. While algorithmic improvements can boost system performance, practical implementations also require hardware optimization and robust signal-processing strategies. In this work, we address these challenges by refining the interrogation mechanism and incorporating a dual interferometric setup. When integrated with a standard demodulation algorithm, this configuration led to a substantial improvement in dynamic range.

Our experimental results show an increase in dynamic range from 125 dB to 139 dB at a 1 kHz acoustic signal frequency without degrading the noise floor of the system. This advancement significantly broadens the practical applicability of underwater acoustic sensing systems, making them more robust and reliable in real-world conditions. In addition to underwater surveillance and marine biology research, such improvements are relevant for environmental monitoring, seismic activity detection, and offshore infrastructure inspection.

## 2. DFB Laser-Based Acoustic Sensing System

**Figure 1:**
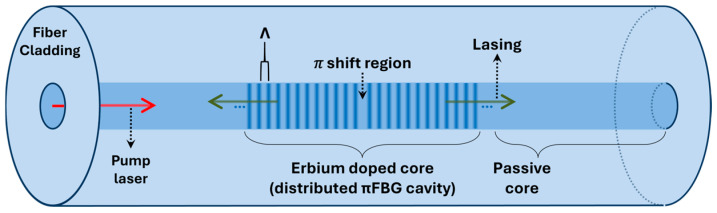
Structure of a DFB fiber laser.

A fiber laser sensor for underwater acoustic sensing features a distributed cavity formed by a π-phase shifted fiber Bragg grating (FBG) written on an active fiber, as shown in Figure 1. The lasing wavelength, $\lambda_{0}$, is given by $\lambda_{0}=2\Lambda n_{\mathrm{eff}}$, where Λ is the grating period (~530 nm at $\lambda_{0}$ = 1550 nm) and $n_{\mathrm{eff}}$ is the effective refractive index of the fiber. The active fiber cavity is doped with erbium ions and is pumped at wavelengths highly absorbed by erbium ions, such as 980 nm or 1480 nm [19,20].

An acoustic signal incident on the fiber cavity deforms its structure by altering both the grating period and the refractive index. This change shifts the lasing wavelength, $\lambda_{0}$ (and thus the frequency, $f_{0}=c/\lambda_{0}$), so the acoustic signal effectively modulates the laser wavelength according to its waveform. The modulation bandwidth depends on the amplitude of the acoustic signal. For example, our measurements indicate that an underwater acoustic projector operating at 1 kHz, located 1 m from the fiber laser sensor, would produce a pressure of approximately 25 Pascals on the sensor spot. This pressure would yield a measured phase shift of about 1.8 radians in an interferometer with a 40 m optical path difference, corresponding to a frequency deviation of roughly 1.5 MHz at 1550 nm and a wavelength deviation of about 12 fm.

### 2.1. Interrogation of Acoustic Induced Frequency Deviations

**Figure 2:**
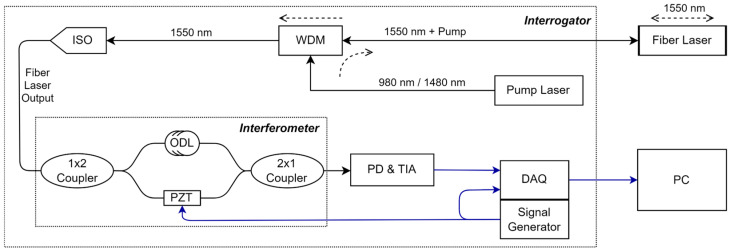
Schematic of the fiber laser interrogator (WDM: wavelength division multiplexer, ISO: isolator, ODL: optical delay line, PZT: piezo fiber stretcher, PD: photodetector, TIA: Trans-Impedance Amplifier, DAQ: data acquisition unit, PC: personal computer).

There are several possible methods to interrogate frequency deviations, such as using a tunable filter or laser where the laser can be scanned to observe a beat frequency or a narrow band filter can be scanned to observe power at filtered bands [13,21]. However, the literature indicates that the most commonly used method for interrogation is a Michelson or Mach–Zehnder interferometer, which converts frequency deviations into phase deviations. In our experiments, a Mach–Zehnder interferometer was employed, since it required fewer components. Figure 2 illustrates the Mach–Zehnder interferometer-based interrogator used to measure the acoustically induced frequency deviations. It is important to note that using a Mach–Zehnder interferometer introduces polarization-related concerns, which can lead to optical power fading, since the interferometer relies on the coherent mixing of two optical signals. This issue can be mitigated by incorporating a polarization controller before the photodetector unit or by adding a polarization beam splitter and using two detectors to measure both polarizations.

The pump laser in the system operates at 976 nm and has a spectral width of about 0.5 nm. It is sent through a wavelength division multiplexer (WDM) to the DFB sensor laser cavity. Depending on the application, there may be a long extension fiber—on the order of kilometers—between the WDM and the fiber laser. The fiber laser has an active (Erbium-doped) area length of ~5 cm and emits in both the forward and backward directions; the backward laser radiation reaching the WDM is directed into an isolator (ISO) to prevent any back reflections before the laser signal enters the interferometer.

The interferometer consists of two 2 × 1 couplers, a PZT (used for phase modulation), and an optical delay line (ODL). The length of the optical delay line is the net delay between the interferometer arms. The first coupler splits the laser output into two paths: one passes through the ODL to provide the required phase difference, and the other passes through the phase modulator (e.g., a PZT stretcher) to impose a desired phase modulation on the laser signal. The PZT (PZ1-SMF4-APC-E, Optiphase, Van Nuys, CA, USA) is driven by the signal generator unit at 36 kHz. The second coupler recombines the two paths and directs the resulting signal into an InGaAs PIN photodetector (PD) with a 100 MHz bandwidth, which converts the optical signal into an electrical current, and then a Trans-Impedance Amplifier (TIA) with a gain factor of 10,000 converts the current to an output voltage. This electrical signal is then acquired by a data acquisition (DAQ) unit. The DAQ unit also receives the PZT driving signal for the phase retrieval algorithm. The DAQ unit acquires data with a 15-bit resolution at 1 MSps for both channels. Finally, the acquired data are transferred to a personal computer (PC) for processing.

### 2.2. Operating Principle of the Interrogator

One of the challenges of using a fiber laser for sensing acoustic signals is that the lasing wavelength is highly sensitive to environmental factors, such as temperature. To mitigate this issue, a method called a phase-generated carrier (PGC) is widely implemented in many applications to eliminate the effects of potential lasing frequency shifts [15].

The PGC method is widely utilized for interrogation, where an acoustically modulated DFB sensor laser signal is phase-modulated using the PZT to isolate it from various environmental effects. To understand the processing of the data, one must first comprehend how frequency deviations are converted into phase deviations. Assuming that the Fiber Laser Output after the isolator is in the form of $2Acos\left(\omega_{0}t\right)$, where $\omega_{0}=2\pi f_{0}$ and $f_{0}=f_{l}+\Delta f_{s}$, $f_{l}$ and $\Delta f_{s}$ represent the lasing frequency and the acoustic signal-induced frequency deviation, respectively.

As shown in Figure 2, the first coupler divides the power into two, resulting in an amplitude of $A\sqrt{2}cos\left(\omega_{0}t\right)$ in each arm. The optical signal in the upper arm is phase-shifted due to the length (L) of the ODL. This phase shift is represented by $\Theta =2\pi n_{\mathrm{eff}}L/\lambda_{0}=\theta_{l}+\theta_{s}$, where $\theta_{l}$ and $\theta_{s}$ correspond to the phase shifts associated with the laser frequency without the acoustic signal and the laser frequency deviation due to the acoustic signal, respectively. Similarly, in the bottom arm, the signal is phase-modulated with a specific voltage, acting as the modulation depth, β, at frequency $f_{c}$, as given in Equations (1) and (2):

(1) $$A\sqrt{2}cos\left(\omega_{0}t+\Theta \right)$$

(2) $$A\sqrt{2}cos\left(\omega_{0}t+\beta cos(\omega_{C}t)\right)$$

The signals are then combined at the second coupler and directed to the PD as given:

(3) $$A\left(cos\left(\omega_{0}t+\Theta \right)+cos\left(\omega_{0}t+\beta cos\left(\omega_{c}t\right)\right)\right).$$

The output of the photodetector is proportional to the intensity of the optical signal, which is also proportional to the square of the amplitude ($I(t)\propto {\left|E\left(t\right)\right|}^{2}$). The high-frequency terms are eliminated by the photodetector, and the DC term is also eliminated, since the TIA employs AC coupling. Assuming the new amplitude can be expressed as B by considering the response of the PD and TIA, the resulting simplified equation is obtained as given:

(4) $$Bcos\left(\beta cos\left(\omega_{c}t\right)+\Theta \right)=X_{j}-Y_{j}.$$

Equation (4) could be further expanded with the help of trigonometric identities and Bessel functions, as given in (5)–(8). It can be seen that two orthogonal signal components are obtained, which eliminates problems such as power fluctuations (if power vanishes in the sine component, it appears in the cosine, and vice versa). From (6) and (8), it can be observed that even harmonics spread out with cos(Θ) and odd harmonics with sin(Θ):

(5) $$X_{j}=Bcos\left(\beta cos\left(\omega_{c}t\right)\right)\cos\left(\Theta \right)$$

(6) $$X_{j}=\left[J_{0}\left(\beta \right)+2\sum_{k=1}^{\infty }J_{2k}\left(\beta \right)\cos\left(2k\omega_{c}t\right)\right]\cos\left(\Theta \right)$$

(7) $$Y_{j}=\mathrm{Bsin}\left(\beta cos\left(\omega_{c}t\right)\right)\sin\left(\Theta \right)$$

(8) $$Y_{j}=\left[2\sum_{k=0}^{\infty }J_{\left(2k+1\right)}(\beta )sin((2k{+1)\omega }_{c}t)\right]\sin\left(\Theta \right)$$

**Figure 3:**
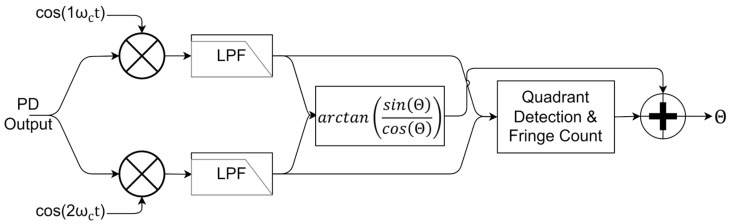
Block diagram of the demodulation algorithm through the PGC arctangent method.

Figure 3 shows a schematic of the arctangent approach to the PGC method, where the phase information is obtained through calculating the arctangent of the two orthogonal components with first and second harmonics, as given in (9)–(12). There are other methods used besides the arctangent approach, such as differential cross-multiplication or orthogonal demodulation [22,23]. However, the arctangent approach stands out as long as the conditions (mentioned next) are met:

(9) $$X_{j}=\cos\Theta \left[J_{0}\left(\beta \right)+2J_{2}\left(\beta \right)\cos\left(2\omega_{c}t\right)+2J_{4}\left(\beta \right)\cos\left(4\omega_{c}t\right)+\ldots \right]$$

(10) $$X_{j}\cos\left(2\omega_{c}t\right)\overset{LPF}{\Rightarrow }\cos\left(\Theta \right)\cdot 2J_{2}(\beta )$$

(11) $$Y_{j}=\sin\left(\Theta \right)\left[{2J}_{1}\left(\beta \right)\cos\left(1\omega_{c}t\right)+{2J}_{3}\left(\beta \right)\cos\left(3\omega_{c}t\right)+\ldots \right]$$

(12) $$Y_{j}\cos\left(\omega_{c}t\right)\overset{LPF}{\Rightarrow }\sin\left(\Theta \right)\cdot 2J_{1}(\beta )$$

After the orthogonal phase terms are acquired, the arctangent can be calculated to obtain the phase information, as given in (13), where this calculation is only valid if $J_{1}\left(\beta \right)$ and $J_{2}\left(\beta \right)$ are equal to each other:

(13) $$\Theta =\arctan\left(\frac{\sin\left(\Theta \right)2J_{1}\left(\beta \right)}{\cos\left(\Theta \right)2J_{2}\left(\beta \right)}\right)\overset{iff}{\Leftrightarrow }J_{1}\left(\beta \right)=J_{2}\left(\beta \right)$$

The Bessel functions of the first kind of order 1 and order 2 first attain equal amplitudes at 2.63 radians. To extract the phase information using the arctangent approach, one must find the phase modulator’s operating point corresponding to a modulation amplitude of 2.63 radians. If the operation point is not correct, the arctangent output will have distortion, which will worsen the results. One method used in this study to approximate this operating point involved scanning the driving amplitude of a PZT stretcher while exposing a fiber laser to a stable sinusoidal acoustic signal or inputting a frequency-modulated laser signal into the system until the minimum distortion value was obtained. In order to find the operating point, a laser source with a constant optical frequency modulation operating at 1 kHz was used, while the PZT driving amplitude was increased with 0.01 V_pp_ steps. At each voltage step, the phase value was derived as described in Figure 3 and the total harmonic distortion was calculated. The minimum total harmonic distortion, which corresponds to the modulation amplitude of 2.63 radians, was obtained at 3.66 V_pp_ for the PZT operating at 36 kHz. The calibration remained stable throughout the experiments. After the arctangent was correctly calculated, the quadrant detection and fringe count algorithms were executed to unwrap the phase, which worked correctly as long as the phase step between the samples did not exceed 2π.

As mentioned before, the acquired phase term ($\Theta =\theta_{l}+\theta_{s}$) was converted from frequency deviations into phase deviations by the interferometer. The phase term contained the laser frequency phase offset, $\theta_{l}$, appearing as a low-frequency component, along with the acoustically derived phase term, $\theta_{s}$. Once the acquired phase term was high-pass-filtered, only the $\theta_{s}$ term remained. Additionally, the phase term included inevitable environmental noise along with the acoustic information; however, the environmental noise was also eliminated using a high-pass filter, as it typically consists of low-frequency components. As a result, the acoustic phase term resulting from the optical phase delay can be unpacked for the Mach–Zehnder interferometer as shown in (14), where L is the physical length of the ODL and v is the speed of light in the medium.

(14) $$\frac{2\pi L}{v}\Delta f_{s}≅\theta_{s}$$

It can be seen from (14) that the resulting phase deviation is a product of the laser’s frequency deviations. Therefore, the laser’s linewidth acts on the measurement results as the frequency noise determining the minimum measurable phase [11]. The lasers used in acoustic sensing are especially designed to have narrow linewidths; in our case, the laser was measured to be less than 5 kHz. The amplitude of the phase term is also directly related to the optical delay line; hence, selection of the ODL length (L) affects the system’s fundamental capabilities.

## 3. Dual Interferometric Approach

Employing a short ODL reduces the frequency-to-phase conversion rate, thereby increasing the upper limit of the measurable frequency deviation while also worsening the minimum measurable frequency deviation. Conversely, a long ODL lowers the maximum measurable frequency deviation but allows for the detection of smaller frequency deviations. To address this trade-off, we propose using dual interferometry, which combines the extended measurement range of a short ODL with the improved sensitivity of a long ODL, providing an enhanced frequency deviation measurement.

### 3.1. System Architecture

**Figure 4:**
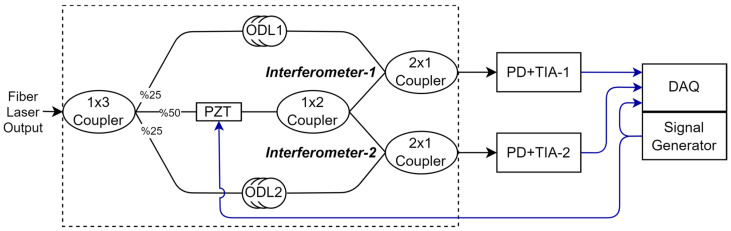
Dual interferometric interrogation schematic based on Mach–Zehnder interferometer (PZT: piezo fiber stretcher, ODL: optical delay line, PD: photodetector, TIA: Trans-Impedance Amplifier, DAQ: data acquisition unit).

Figure 4 shows the proposed dual interferometric interrogation schematics based on the Mach–Zehnder interferometer. The dual interferometric architecture presents a simple approach with few additional components to the single-interferometric architecture. To simplify the schematic shown in Figure 4, the blocks until the Fiber Laser Output in Figure 2 are not drawn, instead inserted as the “Fiber Laser Output”. In the figure, the Fiber Laser Output is divided into three components through the first coupler, with the PZT arm receiving double the power of the ODL arms. This is due to the optical signal being further split into two after the phase modulation. In the Figure 4, the ODL (optical delay line) denotes the individual delay lines inserted in Interferometer−1 and Interferometer−2, whereas the OPD (optical path difference) refers to the total path-length mismatch between the two arms of an interferometer. The optical path difference (OPD) of Interferometer−1 and Interferometer−2 were set to be 2.7 and 155 m, respectively (with the appropriate ODL1 and ODL2). The ratio of the path differences (57 times) was also the phase response ratio for the given frequency shift, as given in Equation (14). As a result, Interferometer−2 was expected to provide 57 times higher amplification of very small acoustic signals, bringing them to a level easily readable by the interrogator. Meanwhile, Interferometer−1, with its lower amplification, was designed to extend the saturation limit for high-amplitude acoustic signals.

### 3.2. Time Domain Measuremements

**Figure 5:**
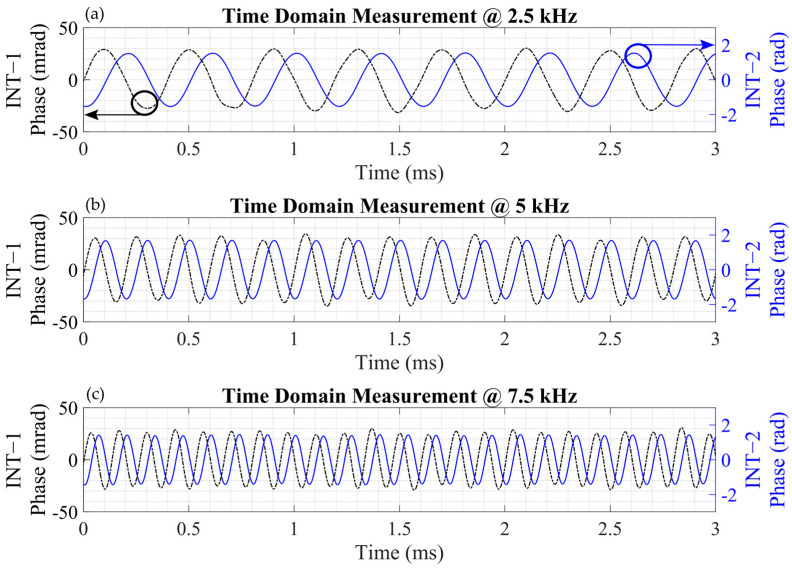
Time-domain signals for 2.5 kHz (**a**), 5 kHz (**b**), and 7.5 kHz (**c**) obtained with Interferometer−1 (INT−1, OPD of ~2.7 m, black) and Interferometer−2 (INT−2, OPD of ~155 m, blue); OPD: optical path difference.

In order to verify the interrogator and the interferometers, the responses of 2.5 kHz, 5 kHz and 7.5 kHz acoustic signals were observed in the time domain. After the demodulation of the acquired signals, the phase information was obtained, as given in Figure 5. The phase amplitudes of the signals from Interferometer−1 (INT−1) and Interferometer−2 (INT−2) for the 2.5 kHz signal are presented in Figure 5a, with values of approximately 0.027 radians for INT−1 and 1.52 radians for INT−2. Figure 5b illustrates the phase amplitudes at 5 kHz, calculated as 0.031 radians for INT−1 and 1.69 radians for INT−2. Lastly, Figure 5c displays the phase amplitudes at 7.5 kHz, which are 0.026 radians for INT−1 and 1.45 radians for INT−2. The ratios of INT−2 to INT−1 at 2.5 kHz, 5 kHz, and 7.5 kHz were calculated to be 56.3, 54.5, and 55.8, respectively—showing good agreement with the expected ratio of the optical path differences.

### 3.3. System Noise Floor Characterization

**Figure 6:**
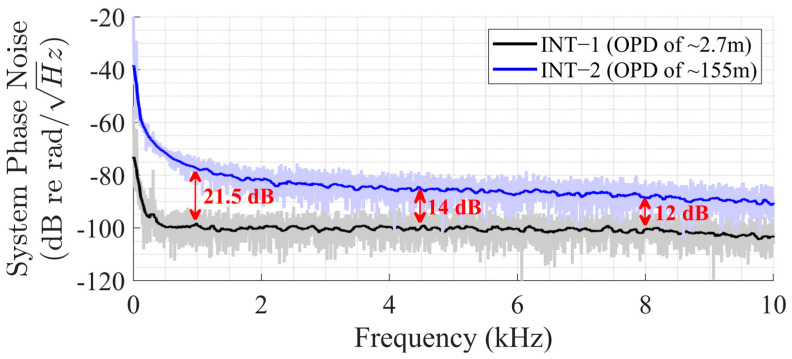
Phase noise measurements of Interferometer−1 (INT−1) and Interferometer−2 (INT−2).

The system noise floors were measured for both interferometers when the DFB sensor laser was active, and the results are shown in Figure 6. The noise floor of Interferometer−1 is higher by 21.5 dB at 1 kHz, 14 dB at 4.5 kHz, and 12 dB at 8 kHz, since the environmental and system frequency noises are also amplified due to the longer optical path. Even though the system noise floor is determined by the DFB sensor laser’s frequency noise and the environmental noise, the dynamic range is related to the interrogator and the demodulation algorithm.

### 3.4. Upper Limit Characterization

To see the limits of the interrogator, the fiber laser was exchanged with a reference laser (Koheras Basik X15, NKT Photonics, Birkerød, Denmark), which had a maximum optical frequency modulation deviation of 500 MHz up to a 20 kHz modulation frequency. The laser could modulate its output frequency, thereby mimicking the DFB sensor laser when exposed to an acoustic signal. The reference laser’s modulation properties were characterized using an interferometer and an RF spectrum analyzer, and the experiments were then conducted accordingly.

**Figure 7:**
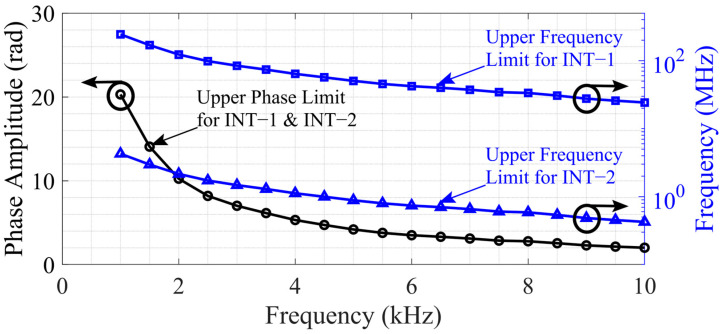
The phase-demodulation upper limits and corresponding frequency-demodulation upper limits of Interferometer−1 (INT−1) and Interferometer−2 (INT−2).

To characterize the interrogator’s response to the modulation frequency, the reference laser was swept from 1 kHz to 10 kHz in 500 Hz increments. For each modulation frequency, the upper limit was determined by increasing the frequency deviation until the phase information could no longer be unwrapped or when the total harmonic distortion exceeded −25 dB. The phase detection limit occurred when the phase step between the measurement samples exceeded 2π, resulting in ambiguity and preventing the algorithm from unwrapping the phase correctly. The upper limit in terms of phase was independent of the optical path differences of the interferometers, as it was mainly limited by the phase extraction resolution, which was bound to the PZT driving frequency and the ability of the algorithm. The phase limit was measured by Interferometer−2 (as it reached the phase limit with much smaller frequency deviations) and is shown in Figure 7. For instance, at 1 kHz, Interferometer−2 reaches an upper phase amplitude limit of about 20 radians at a laser frequency deviation of roughly 4.2 MHz. Meanwhile, Interferometer−1 has a gain factor that is 57 times lower, although it is still linear. Hence, to reach the same ~20-radian limit at 1 kHz for Interferometer−1, the laser frequency deviation would need to be around 240 MHz.

### 3.5. Results

**Figure 8:**
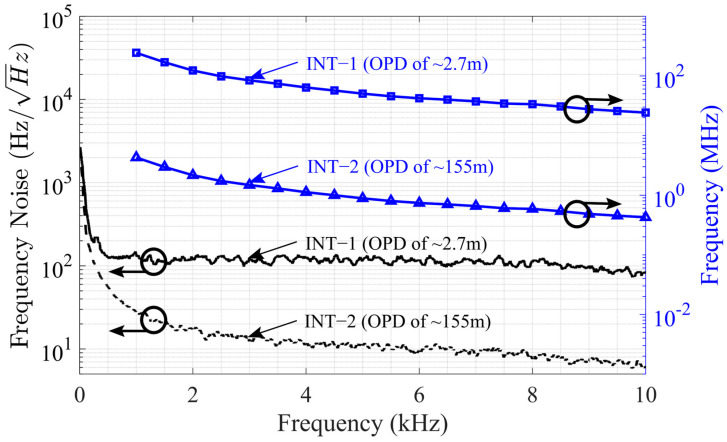
Frequency noise and frequency upper limits of Interferometer−1 (INT−1) and Interferometer−2 (INT−2).

**Figure 9:**
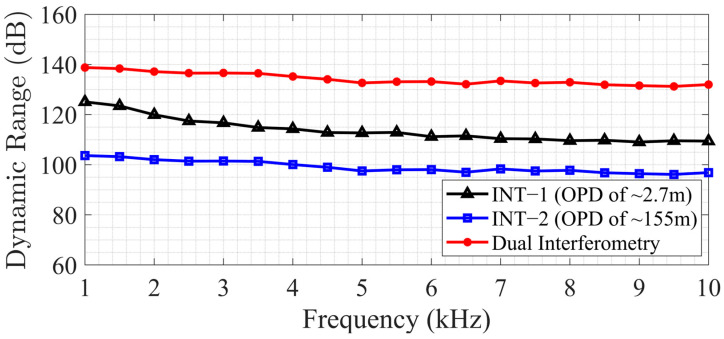
Dynamic ranges obtained with Interferometer−1 (INT−1), Interferometer−2 (INT−2), and dual interferometry.

Figure 8 shows the frequency noise and the upper limits obtained from Figure 6 and Figure 7 using the equation given in (14). The frequency noise was calculated to be $\sim 136\;Hz/\sqrt{Hz}$ for INT−1 and $\sim 28\;Hz/\sqrt{Hz}$ for INT−2 at 1 kHz, reaching down to $\sim 82\;Hz/\sqrt{Hz}$ for INT−1 and $\sim 6\;Hz/\sqrt{Hz}$ for INT−2 at 10 kHz. The upper limit for INT−1 starts at approximately 4.2 MHz at 1 kHz and decreases to around 0.4 MHz. Similarly, for INT−2, the upper limit starts at approximately 240 MHz and decreases to 24 MHz at 10 kHz. The dynamic range of the dual interferometric interrogator was calculated by the ratio of the upper limit to the noise floor and is shown in Figure 9. It can be observed that the dynamic range declines with increasing frequency because the phase changes more rapidly at higher modulation frequencies, making it difficult for the algorithm to correctly unwrap the phase. Interferometer−2 has a dynamic range starting at 103 dB at 1 kHz and ending at 97 dB at 10 kHz. Interferometer−1 has a higher dynamic range due to the advantage of lower system phase noise, starting with 125 dB at 1 kHz and reaching down to 109 dB at 10 kHz. The overall combined dynamic range was derived from Figure 8 by using the lower frequency noise and higher upper limit. It was calculated to be 139 dB at 1 kHz and decreased to 132 dB at 10 kHz, exhibiting a more linear trend compared with the dynamic ranges of INT−1 and INT−2. The combined dynamic range was achieved by utilizing both interferometers to obtain two distinct phase measurements, with the appropriate result selected based on the measurement conditions. Rather than applying direct signal fusion, a selection-based strategy was employed: the high-sensitivity interferometer was used for detecting small signal variations, while the long-OPD interferometer was utilized to extend the dynamic range and resolve phase ambiguities.

### 3.6. Comparison

In light of these findings, it is useful to compare them with the results from similar system configurations in the literature, which highlight both consistent trends and variations in dynamic range performance. A simulation-based study employing a Mach–Zehnder interferometer, combined with phase-generated carrier (PGC) arctangent and differential-cross-multiplication demodulation techniques, reported dynamic ranges just below 80 dB and up to 120 dB across the 10 Hz to 5 kHz frequency range, respectively [18]. Similar experimental systems have also been investigated in several previous studies. In one such study, a Michelson interferometer equipped with a PZT module operating at 10 kHz and an interferometric optical path difference (OPD) of 10 m was implemented. This configuration demonstrated a dynamic frequency range spanning from 20 Hz to 2 kHz, achieving a peak dynamic range of 120 dB at 63 Hz, which gradually decreased to below 100 dB at 1 kHz [13]. Another study proposed a comparable Michelson interferometer setup, reporting dynamic ranges of 115 dB at 10 Hz and 98 dB at 100 Hz and declining to 80 dB at 400 Hz [24].

## 4. Conclusions

This study focuses on enhancing underwater acoustic sensing system capabilities through using dual interferometry. This research highlights the limitations of conventional piezoelectric-based methods, including their susceptibility to environmental factors and limited dynamic ranges and response times. By utilizing fiber-optic hydrophones, particularly DFB sensor laser-based systems, these challenges can be mitigated due to their simpler structures, high durability, and broad detection capabilities.

A dual-interferometry setup employing Mach–Zehnder interferometers was developed to improve the phase measurements and extend the system’s dynamic range. The implementation of the dual interferometric approach demonstrated significant enhancements, achieving an extended dynamic range of 139 dB at 1 kHz, exceeding the previously obtained threshold of 125 dB without degrading the noise floor. While longer optical paths amplified the signals, they also introduced higher noise levels and upper limit limitations. The experimental measurements were taken with the dual interferometric setup, and the system was validated with a reference laser, which confirmed the system’s capacity to detect acoustic signals across a broad frequency range while maintaining a high resolution and sensitivity. In summary, this study establishes the feasibility and effectiveness of using a DFB sensor laser with dual interferometric interrogation to achieve superior dynamic range, sensitivity, and adaptability in underwater acoustic sensing applications.

## Data Availability

Data are available upon request.
